# The Role of Estrogen across Multiple Disease Mechanisms

**DOI:** 10.3390/cimb46080483

**Published:** 2024-07-29

**Authors:** Xiuting Xiang, Praneetha Palasuberniam, Rahmawati Pare

**Affiliations:** Department of Biomedical Science, Faculty of Medicine and Health Sciences, Universiti Malaysia Sabah, Kota Kinabalu 88400, Malaysiapraneetha@ums.edu.my (P.P.)

**Keywords:** estrogen, mechanism, multiple disease, Alzheimer’s disease, depression, cardiovascular disease, diabetes, osteoporosis, gastrointestinal diseases, estrogen-dependent cancers

## Abstract

Estrogen is a significant hormone that is involved in a multitude of physiological and pathological processes. In addition to its pivotal role in the reproductive system, estrogen is also implicated in the pathogenesis of a multitude of diseases. Nevertheless, previous research on the role of estrogen in a multitude of diseases, including Alzheimer’s disease, depression, cardiovascular disease, diabetes, osteoporosis, gastrointestinal diseases, and estrogen-dependent cancers, has concentrated on a single disease area, resulting in a lack of comprehensive understanding of cross-disease mechanisms. This has brought some challenges to the current treatment methods for these diseases, because estrogen as a potential therapeutic tool has not yet fully developed its potential. Therefore, this review aims to comprehensively explore the mechanism of estrogen in these seven types of diseases. The objective of this study is to describe the relationship between each disease and estrogen, including the ways in which estrogen participates in regulating disease mechanisms, and to outline the efficacy of estrogen in treating these diseases in clinical practice. By studying the role of estrogen in a variety of disease mechanisms, it is hoped that a more accurate theoretical basis and clinical guidance for future treatment strategies will be provided, thus promoting the effective management and treatment of these diseases.

## 1. Introduction

Estrogen is an endogenous hormone that plays a variety of important physiological and pathological functions in the human body [1]. In addition to its role in regulating the development and function of the reproductive system, estrogen plays a pivotal role in the normal functioning of numerous other organs and systems. These include the nervous, endocrine, cardiovascular, skeletal, digestive, and even some cancerous systems [2]. A substantial body of research has demonstrated that a decline in estrogen levels can precipitate the onset of Alzheimer’s disease [3], depression [4], diabetes [5], cardiovascular disease [6], osteoporosis [7], and gastrointestinal diseases [8]. Conversely, estrogen also plays a significant role in the pathogenesis of estrogen-dependent cancers [9]. For menopausal women, the decline in estrogen levels is accompanied by a range of physiological and psychological challenges. Although numerous studies have investigated the mechanism of estrogen on a specific disease, the overall effect of estrogen on disease mechanisms remains poorly understood. This review aims to comprehensively explore the ways in which estrogen participates in regulating the mechanisms of Alzheimer’s disease, depression, cardiovascular disease, diabetes, osteoporosis, gastrointestinal diseases, and estrogen-dependent cancers. Additionally, it will outline the efficacy of estrogen treatment in clinical trials and provide prospects for future research and treatment strategies. In this way, we aim to gain a comprehensive understanding of the role of estrogen in a variety of diseases, and to provide new ideas and guidance for the treatment and management of related diseases.

## 2. Alzheimer’s Disease

### 2.1. Estrogen and Alzheimer’s Disease

Alzheimer’s disease (AD) is a chronic neurodegenerative disease that primarily affects older adults and manifests as progressive cognitive decline. However, recent research has highlighted the crucial role of estrogen in influencing the risk of AD, particularly in relation to age [10]. A significant association between estrogen deficiency and increased susceptibility to Alzheimer’s disease has been reported in numerous studies [11,12]. Specifically, postmenopausal women who experience a significant decline in estrogen levels have an increased risk of developing AD compared to their premenopausal counterparts [13]. This observation underscores a strong relationship between estrogen status and Alzheimer’s disease incidence. In addition, epidemiologic data support the notion that women entering menopause have a 1.67-fold increased risk of developing AD [14]. This statistically significant increase in risk underscores the potential impact of estrogen depletion on AD pathogenesis.

### 2.2. Estrogen’s Impact on Alzheimer’s Disease Mechanisms

**Figure 1:**
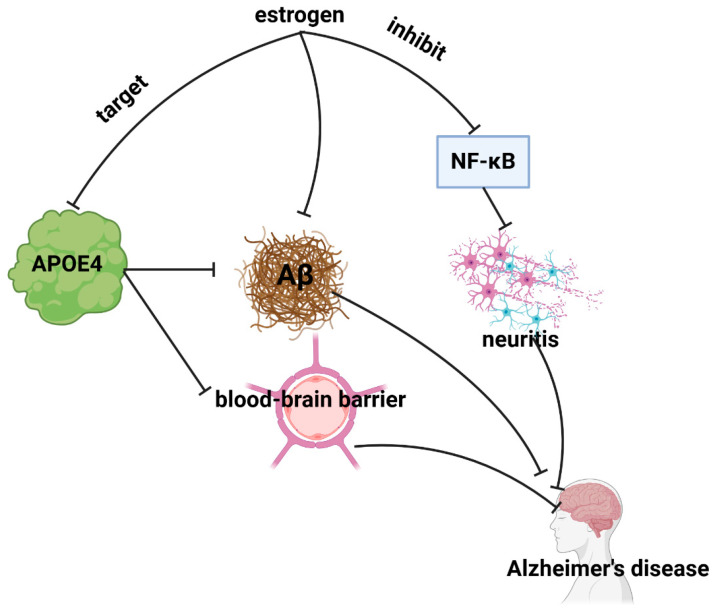
Diagram of the mechanism by which estrogen improves Alzheimer’s disease. APOE4, apolipoprotein E4; Aβ, amyloid-beta; NF-κB, NF-nuclear factor κ-B; blunt arrows (┴), inhibit.

The primary pathogenesis of AD involves the formation of β-amyloid (Aβ) plaques, disruptions in gene trafficking, and the presence of inflammation [15,16,17]. Emerging research suggests that estrogen may have potential in delaying or preventing the onset of Alzheimer’s disease. This is supported by evidence outlined in Figure 1, illustrating mechanisms through which estrogen may exert its protective effects.

#### 2.2.1. Targeting and Inhibition of APOE4 Gene

The apolipoprotein E4 (APOE 4) genotype is widely acknowledged as the primary genetic factor contributing to cognitive decline and an elevated risk of Alzheimer’s disease (AD) [18]. Estrogen, acting as a selective ligand for the APOE4 gene, has been demonstrated to target APOE4 [19,20], thereby reducing amyloid-beta (Aβ) deposition [21], enhancing Aβ clearance in the brain, and mitigating blood–brain barrier damage [22]. These actions indicate the potential for alleviating symptoms associated with Alzheimer’s disease. Furthermore, the interaction between APOE and estrogen receptors (ERs) may exert an influence on Alzheimer’s-related outcomes. It is postulated that disparate ERs binding to APOE may result in disparate biological effects. For example, the activation of ERβ has been associated with a reduction in APOE mRNA expression [23] and an increase in synaptic protein expression [24]. Conversely, evidence suggests that the activation of ERα may result in an elevation of APOE levels, although the specific mechanisms remain unclear [23]. It is a consistent finding that individuals with AD typically exhibit elevated levels of ERα [25]. A comprehensive investigation is required to gain a full understanding of the intricacies of these interactions and their precise roles in disease progression.

#### 2.2.2. Enhanced Clearance of Aβ Accumulation

Research has indicated that estrogen facilitates the removal of Aβ from the brain [11,26], potentially through various mechanisms. These include enhancing the activity of enzymes responsible for Aβ degradation, such as insulin-degrading enzymes and enkephalins [27,28], and bolstering the function of brain cells involved in Aβ clearance, such as glial cells [29]. Additionally, estrogen may impact Aβ accumulation and clearance by regulating the expression of proteins associated with Aβ metabolism, such as annexin A1 (AnxA1) [30], and by stimulating the α-secretase pathway to inhibit Aβ generation [31].

#### 2.2.3. Anti-Inflammatory Effects on Neuroinflammation

The anti-inflammatory effects of estrogen on neuroinflammation play a critical role in the prevention and treatment of Alzheimer’s disease. Neuroinflammation, characterized by the brain’s inflammatory response to injury or infection, is initially a protective mechanism [32]. However, its persistence may contribute to neurodegeneration and the development of AD [33]. A key player in neuroinflammation is nuclear factor κ-B (NF-κB), which is primarily found in an inactive state in the cytoplasm [34]. Sustained activation of NF-κB is associated with AD pathology [35,36]. Studies have shown that estrogen supplementation has beneficial effects in reducing neuroinflammation by targeting NF-κB [32]. Research suggests that estrogen may reduce NF-κB activity [3], particularly in astrocytes exposed to Aβ [3]. In addition, estrogen has been found to inhibit NF-κB translocation to the nucleus, thereby exerting anti-inflammatory effects on microglia, another type of brain cell involved in neuroinflammation [37].

In conclusion, the role of estrogen in AD pathogenesis involves targeting the APOE4 gene, enhancing Aβ clearance, and suppressing neuroinflammation. These mechanisms offer potential therapeutic avenues for the prevention and treatment of AD.

### 2.3. Clinical Findings on the Efficacy of Estrogen in the Treatment of Alzheimer’s Disease

Although estrogen has shown potential efficacy in attenuating the mechanisms of AD, results from clinical trials have been conflicting. Clinical trials have examined the effects of estrogen therapy in patients with AD with mixed results. Preliminary evidence from clinical trials indicates that estrogen therapy may offer some benefits, including improvements in cognitive function and the slowing of disease progression [38,39,40]. Others have been unable to demonstrate a significant degree of efficacy, and in some cases, have even raised concerns about the potential for adverse effects [41,42]. One of the most notable clinical trials is the Women’s Health Initiative Memory Study (WHIMS). The study, which included postmenopausal women aged 65 and older, found that estrogen therapy, especially when combined with progestin, was associated with an increased risk of dementia and cognitive decline [43]. Consequently, no definitive guidelines or standard protocols for estrogen therapy for AD have been established.

## 3. Depression

### 3.1. Estrogen and Depression

Depressive episodes frequently manifest as more prolonged and recurrent in women compared to men, particularly during the perimenopausal and hypoestrogenic phases [44]. During this period, the decline in estrogen levels is accompanied by dysfunctions in the hypothalamic–pituitary–adrenal (HPA) axis, resulting in impaired mood and cognitive functions. Additionally, there is a significant reduction in hippocampal volume and activity in the hippocampal region, which further elevates the risk of depression [45]. Moreover, epidemiological evidence supports the notion that women undergo an increased risk of depression during the transition to menopause, with a significantly higher incidence compared to premenopausal stages [46]. These findings highlight the significant impact of estrogen on depression.

### 3.2. Estrogen’s Impact on Depression Mechanisms

**Figure 2:**
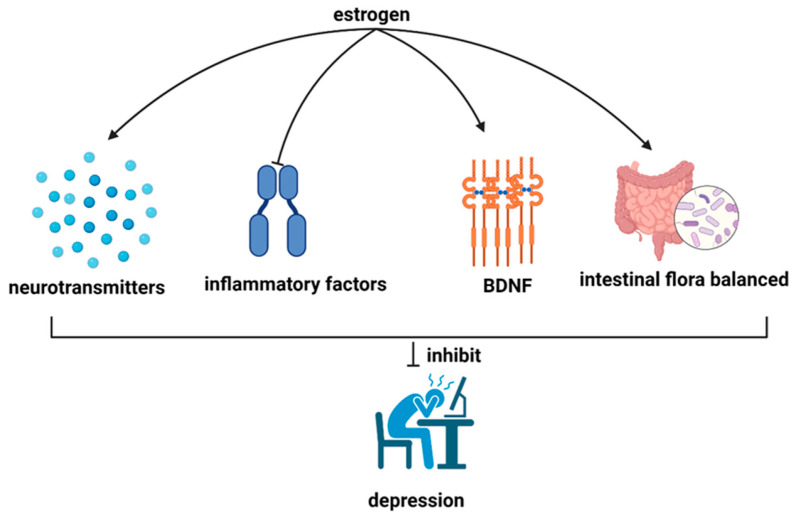
Diagram illustrating the mechanisms by which estrogen relieves depression. Neurotransmitters: including 5-HT (5-hydroxytryptamine), NA (norepinephrine), DA (dopamine), Glu (glutamate), and GABA (gamma-aminobutyric acid); inflammatory factors: including such as CRP, IL-6, and TNF-alpha; BDNF, brain-derived neurotrophic factor; sharp arrows (→), stimulate; blunt arrows (┴), inhibit.

In recent years, numerous scholars have provided evidence supporting the antidepressant effects of estrogen in menopausal women experiencing depression [47,48,49,50,51,52,53]. This is supported by evidence outlined in Figure 2, illustrating mechanisms through which estrogen may exert its protective effects.

#### 3.2.1. Regulation of Neurotransmitter Release

The central role of estrogen in alleviating depressive symptoms is closely linked to its regulation of neurotransmitter release. As a potent modulator in the brain, estrogen influences the secretion and activity of neurotransmitters, the chemical messengers essential for inter-neuronal communication [54]. By fine-tuning the levels of neurotransmitters such as 5-HT (5-hydroxytryptamine), NA (norepinephrine), DA (dopamine), Glu (glutamate), and GABA (gamma-aminobutyric acid), estrogen orchestrates a delicate balance critical for mood regulation and emotional well-being [55,56,57]. Consequently, estrogen’s capacity to regulate neurotransmitter release is becoming increasingly evident as a pivotal mechanism underlying its antidepressant effects. Nevertheless, the precise pathways and mechanisms of action require further investigation, and future studies could prioritize addressing this knowledge gap.

#### 3.2.2. Reduction in Inflammatory Factors

Another mechanism by which estrogen alleviates depression is by reducing the secretion of inflammatory factors. It is becoming increasingly clear that inflammation plays a central role in the etiology of depression. Elevated levels of inflammatory markers are often observed in individuals with depressive symptoms [58]. Estrogen exerts its anti-inflammatory effects by inhibiting the production and release of inflammatory molecules in the body [59]. By reducing the inflammatory response, estrogen helps protect the integrity of nerve cells and prevents the damaging effects of prolonged inflammation on the central nervous system [60,61]. Conversely, estrogen deficiency can lead to increased inflammatory activity, which can exacerbate the onset and severity of depressive symptoms [61,62,63]. Therefore, estrogen’s ability to attenuate inflammatory pathways is an important mechanism by which it exerts its antidepressant effects.

#### 3.2.3. Regulation of Brain-Derived Neurotrophic Factor

Estrogen’s influence extends to its regulatory effect on brain-derived neurotrophic factor (BDNF), a key player in brain neuroplasticity and mood regulation. BDNF is a neurotrophin that supports the survival, growth, and differentiation of neurons, which is critical for maintaining optimal brain function [64]. Research has consistently shown a strong association between reduced BDNF levels and the development of depression [65,66]. Estrogen modulates BDNF expression, promoting its synthesis and release in the central nervous system [67,68]. This upregulation of BDNF levels facilitates neurogenesis, synaptic plasticity, and neuronal survival, all of which contribute to improved mood and resilience to depressive symptoms [69]. Conversely, estrogen depletion or deficiency leads to decreased BDNF expression, which impairs the brain’s ability to adapt and respond to stressors, thereby increasing susceptibility to depression [70]. Thus, estrogen’s regulatory effect on BDNF represents a critical mechanism by which it exerts its antidepressant properties.

#### 3.2.4. Maintenance of Gut Flora Balance

In addition to its central effects, estrogen can impact depressive behavior by modulating disruptions in gut flora. Research suggests that alterations in gut microbiota composition, often observed during hormonal fluctuations, are linked to changes in mood and behavior [71]. A bidirectional relationship has been identified between estrogen and gut flora, with gut microbiota influencing estrogen levels and estrogen, in turn, promoting the abundance of beneficial flora [72]. Through this reciprocal relationship, depressive behaviors can be modulated [73,74]. However, there is a paucity of research in the literature on the specific pathways involved. Nevertheless, this highlights the potential of interventions targeting gut microbiota modulation to complement traditional antidepressant therapies in managing depression associated with hormonal changes. 

In conclusion, estrogen is likely to contribute to the development of depression through several mechanisms. These include the promotion of neurotransmitter release, the upregulation of BDNF expression, the maintenance of gut flora balance, and the suppression of inflammatory factors.

### 3.3. Clinical Research Findings on Estrogen Therapy for Depression

Several clinical investigations have been undertaken to assess the effectiveness of estrogen therapy for alleviating depression symptoms in menopausal women. Nevertheless, the clinical outcomes regarding estrogen’s efficacy in depression treatment have exhibited inconsistency. While some trials have demonstrated positive results, such as reduced depressive symptoms with topical hormone therapy or transdermal estrogen supplementation in perimenopausal women [75,76,77,78], others have not observed significant improvements, particularly in postmenopausal women [79]. Currently, estrogen therapy is not a recommended primary treatment for menopausal depression, with traditional antidepressants, psychotherapy, and lifestyle modifications being the preferred approaches [80]. Further research is necessary to elucidate the optimal utilization of estrogen therapy in the management of depression during menopause and its potential combined effects with other treatments.

## 4. Diabetes

### 4.1. Estrogen and Diabetes

The role of estrogen in metabolic regulation within the body is of pivotal importance. The relationship between estrogen and diabetes mellitus has been extensively documented in the literature [81]. A significant association has been identified between declining estrogen levels in women and an elevated risk of diabetes mellitus [82,83]. For example, women frequently experience a decline in estrogen levels during menopause or following a hysterectomy, which may result in insulin resistance and subsequently elevate the risk of diabetes [84,85]. Moreover, estrogen plays a pivotal role in the distribution of adipose tissue, insulin secretion and utilization, and blood glucose regulation. A deficiency in this hormone may impede these processes, thereby accelerating the development of diabetes [86]. Consequently, it is of the utmost importance to maintain optimal estrogen levels to prevent and manage diabetes mellitus.

### 4.2. Estrogen’s Impact on Diabetes

**Figure 3:**
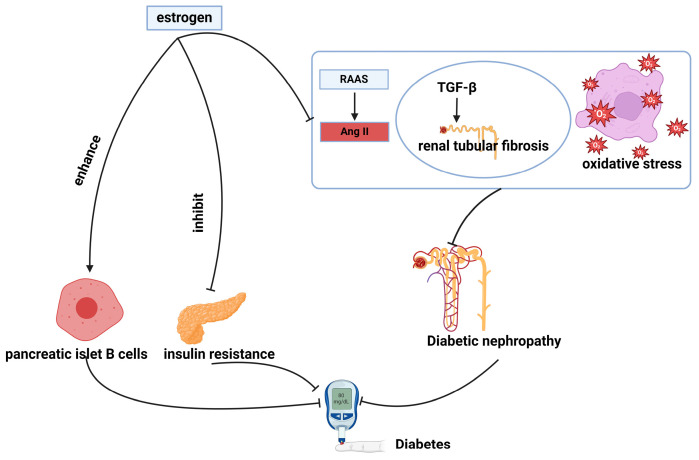
Diagram of the mechanism of action of estrogen in the improvement of diabetes. RAAS: renin–angiotensin–aldosterone system; Ang II: angiotensin II. Sharp arrows (→), stimulate; blunt arrows (┴), inhibit.

Estrogen plays a pivotal role in the pathogenesis of diabetes, with recent findings suggesting its involvement in improving glucose metabolism through several pathways, as illustrated in Figure 3.

#### 4.2.1. Enhancement of Pancreatic Islet B Cell Function

Estrogen plays a critical role in maintaining and enhancing the functionality of pancreatic islet β-cells by primarily inhibiting their apoptotic processes. Activation of ERα, ERβ, or GPER by estrogen protects pancreatic β-cells from apoptosis. The activation of ERα by estrogen has been demonstrated to support mitochondrial dynamics and endoplasmic reticulum–Golgi function. This is achieved by inhibiting Oma1 and Chop, which, in turn, promotes β-cell survival and insulin secretion [87]. Recent research also indicates that estrogen, ERβ, and GPER play a substantial role in protecting pancreatic β-cells from apoptosis. The formation of dimers from ERα and ERβ binding serves to further enhance this anti-apoptotic effect; however, it can be inhibited by the GPER antagonist G1 [88]. In terms of the stress response of pancreatic cells, estrogen may protect pancreatic β-cells by suppressing apoptosis-induced endoplasmic reticulum (UPR) stress and enhancing adaptive UPR activation [89]. Furthermore, under conditions of high glucose, estrogen reduces apoptosis in pancreatic β-cells by inhibiting the expression of apoptosis-related proteins such as B-cell translocator gene 2 (BTG2), p53, and bcl-2-associated X protein (Bax) [90]. Moreover, inflammation is recognized as a significant factor in the pathogenesis of pancreatic β-cell dysfunction [91]. Given estrogen’s known anti-inflammatory properties, it is plausible that estrogen contributes to maintaining pancreatic β-cell function by reducing inflammation.

#### 4.2.2. Alleviating Insulin Resistance

Estrogen improves glucose metabolism by alleviating insulin resistance. Studies consistently show that estrogen reduces the risk of insulin resistance in menopausal women, thus decreasing the incidence of diabetes [92,93]. Supporting this, research on mice subjected to bilateral ovariectomy reveals a higher susceptibility to insulin resistance compared to normal mice [94]. Estrogen regulates insulin resistance through various mechanisms. Firstly, it may influence the expression and function of insulin receptors, potentially enhancing cellular sensitivity to insulin. This effect could occur by controlling the expression of insulin receptor substrate-1, thereby amplifying signals through the insulin-like growth factor signaling pathway [95]. Secondly, estrogen can modulate the distribution and metabolism of adipose tissue [96], reducing fat accumulation in unconventional sites and lowering lipid levels in the blood, consequently alleviating insulin resistance [97]. Additionally, estrogen affects glucose uptake and utilization in muscle tissue, increasing the efficiency of glucose utilization and aiding in the improvement of insulin resistance [98].

#### 4.2.3. Counteracting Diabetic Nephropathy

Estrogen may counteract diabetic nephropathy through three potential mechanisms. Firstly, it regulates the renin–angiotensin–aldosterone system (RAAS), vital for blood pressure and fluid balance, which is activated by the kidney, primarily through angiotensin II (Ang II) [99,100,101,102]. Estrogen has been found to attenuate Ang II-induced renal insufficiency by upregulating endothelial nitric oxide synthase (eNOS) expression [103,104] and accelerating nitric oxide (NO) release, which promotes vasodilation and regulates vascular tone [105]. Secondly, estrogen has been demonstrated to improve renal tubular fibrosis, a characteristic feature of diabetic kidneys driven by increased transforming growth factor-beta (TGF-β) activity, leading to excessive production of extracellular matrix proteins [106]. Estrogen disrupts TGF-β expression and its downstream signaling pathways mediated by Smad proteins such as Smad2, Smad3, Smad6, and Smad7 [107,108], thereby reducing the production of connective tissue growth factor (CTGF) and inhibiting the progression of renal tubular fibrosis [109]. Additionally, estrogen indirectly regulates TGF-β levels by modulating macrophage infiltration into the kidney, as macrophages are the primary source of TGF-β in diabetic nephropathy [110]. Finally, estrogen exerts a protective effect on the kidney by reducing oxidative stress. Estrogen inactivates nitrogen oxides (NOx), inhibits superoxide anion production, and diminishes oxidative stress in the kidney, thus mitigating renal injury [105]. These three mechanisms collectively demonstrate estrogen’s potential in ameliorating diabetic nephropathy and preserving renal function.

In summary, estrogen exerts a protective effect on the development of diabetes mellitus by safeguarding the function of pancreatic islet B cells, impeding the resistance of islet cells, and inhibiting the progression of diabetic nephropathy (this encompasses the inhibition of the RAAS, the prevention of tubular fibrosis, and the mitigation of oxidative stress).

### 4.3. Clinical Research Findings on Estrogen Therapy for Diabetes

Clinical research findings on estrogen therapy for diabetes have shown promising outcomes. Studies indicate that estrogen therapy can significantly improve the condition of diabetic patients [111,112,113]. Women undergoing estrogen therapy exhibit higher insulin sensitivity [114], improved glucose homeostasis [115], and lower levels of glycated hemoglobin (HbA1c) [116] compared to those not receiving treatment. These findings suggest that estrogen therapy may serve as an effective intervention to enhance diabetes management particularly for women in perimenopause.

## 5. Cardiovascular Disease

### 5.1. Estrogen and Cardiovascular

It is well established that estrogen exerts a significant influence on cardiovascular health, with its role in metabolic regulation being pivotal [117,118]. It has been demonstrated that women are typically diagnosed with cardiovascular disease (CVD) approximately 10 years later than men due to the protective effect of estrogen. However, following menopause, women are at an elevated risk of developing CVD, irrespective of age and other cardiovascular risk factors [119]. Consequently, it is of paramount importance to maintain optimal estrogen levels in order to preserve cardiovascular health and to mitigate the risk of cardiovascular diseases.

### 5.2. Estrogen’s Impact on Cardiovascular Disease

**Figure 4:**
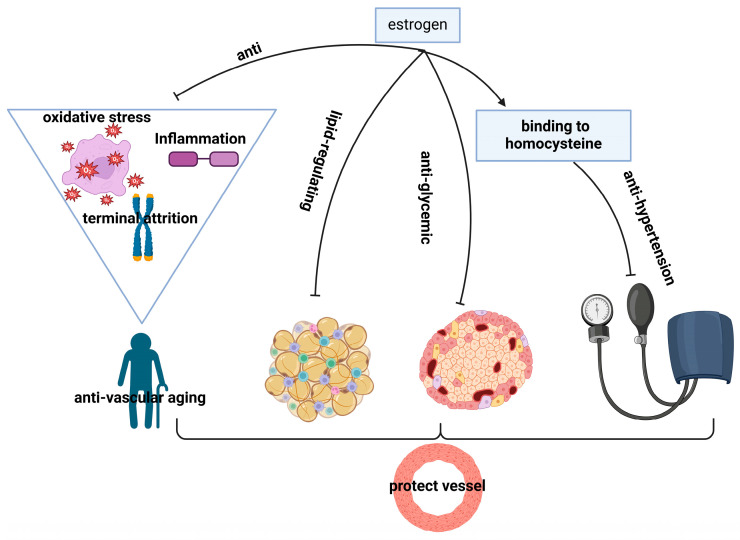
Diagram of the mechanism of action of estrogen in the protection of blood vessels. HYC: homocysteine; sharp arrows (→), stimulate; blunt arrows (┴), inhibit.

A growing body of experimental evidence supports the cardiovascular protective effects of estrogen. These effects are mediated through various mechanisms (Figure 4).

#### 5.2.1. Anti-Vascular Aging Effects

Vascular aging is defined as a progressive decline in endothelial function, increased vascular remodeling, inflammation, and arterial stiffness [120]. Oxidative stress plays an important role in vascular aging, and estrogen can counteract it through a multitude of mechanisms. Estrogens exert antioxidant effects by increasing nitric oxide (NO) bioavailability via cyclooxygenase (COX)-mediated superoxide production, modulating reactive oxygen species (ROS), upregulating Cu/Zn superoxide dismutase expression, or modulating the estrogen receptor beta (ERβ) to estrogen receptor alpha (ERα) ratio [121,122,123]. Inflammation also plays a crucial role in vascular senescence, with chronic inflammation being a significant risk factor for cardiovascular diseases (CVDs). Inflammatory molecules such as chemokines, transcription factors, adhesion molecules, interleukins, and vascular endothelial growth factor (VEGF) contribute to this process [124]. It has been demonstrated that estrogen can inhibit the expression of pro-inflammatory mediators, thereby reducing inflammation [125]. It is notable that the estrogen-mediated reduction in the inflammatory response to C-reactive protein (CRP) is observed primarily in young and non-aged female cells [126]. This is in contrast to menopausal women, who undergo a transition from an anti-inflammatory state to a pro-inflammatory state with age [127]. Furthermore, estrogen combats vascular senescence by addressing anti-terminal attrition. Telomere length, closely associated with human telomerase reverse transcriptase (hTERT), is significantly lower in patients with coronary artery disease compared to healthy individuals of the same age group [128,129]. A study indicated that supplementing estrogen to mice over a 3-week period restores diminished estrogen levels, leading to heightened expression of the TERT gene and increased telomerase activity [130]. Additionally, resveratrol, a phytoestrogen, exhibits varying degrees of estrogen receptor agonism across different experimental models [131]. It has been noted for its ability to activate phosphatidylinositol 3-kinase (PI3-K)/Akt signaling, thereby enhancing telomerase activity and consequently retarding the aging process of endothelial progenitor cells [132]. This multifaceted approach highlights estrogen’s potential in mitigating vascular aging and its associated complications.

#### 5.2.2. Regulation of Lipid

Estrogen exerts a regulatory effect on lipids, thereby conferring protection on blood vessels. It is well established that abnormal lipid metabolism represents a significant risk factor for cardiovascular disease. In menopausal women, alterations in estrogen secretion may result in disturbances to lipid metabolism [133]. Studies have indicated that estrogen has the potential to beneficially impact lipid profiles by increasing levels of high-density lipoprotein (HDL) and decreasing levels of low-density lipoprotein (LDL) [134,135,136]. Moreover, estrogen regulates energy intake and influences the secretion of adipokines, such as leptin and adiponectin, which play a vital role in lipid metabolism [137]. Furthermore, estrogen participates in lipid metabolism across multiple tissues, including adipose tissue, liver, and skeletal muscle [138,139,140,141]. These multiple actions underscore estrogen’s potential to maintain lipid homeostasis and protect blood vessels from the detrimental effects of abnormal lipid metabolism, thereby contributing to cardiovascular health.

#### 5.2.3. Regulation of Glycemia

Estrogen exerts a protective effect on blood vessels by regulating glycemia. Endothelial dysfunction represents a significant contributor to cardiovascular complications in diabetes. Hyperglycemia, a hallmark of diabetes, exacerbates vascular endothelial dysfunction by promoting apoptosis and inhibiting vascular endothelial function [142,143]. Estrogen, as discussed in the diabetes section, has been shown to intervene in the development of diabetes. This indicates that estrogen may have a potential role in mitigating endothelial dysfunction associated with diabetes, thereby offering cardiovascular protection in diabetic individuals. 

#### 5.2.4. Antihypertension

It has been postulated that estrogen may protect blood vessels due to its antihypertensive properties. Clinical studies have demonstrated a significant correlation between estrogen levels and the prevalence of hypertension, particularly in individuals with elevated systolic blood pressure. In perimenopausal and menopausal women, systolic blood pressure is observed to be approximately 4–5 mmHg higher compared to premenopausal women, with an annual increase of approximately 5 mmHg [144]. It has been postulated that estrogen may regulate Hcy levels through the formation of a complex with homocysteine (Hcy) [145]. This complex has been demonstrated to alleviate hypertension by inhibiting the secretion of pro-inflammatory factors, including monocyte chemotactic protein 1 (MCP-1) and interleukin-8 (IL-8), in human monocytes [127,146]. Furthermore, estrogen stimulates the production of endogenous hydrogen sulfide (H2S), a potent vasodilator [147], ameliorates insulin resistance [148], and suppresses the excitability of the sympathetic nervous system (SNS) [149] to exert its blood-pressure-lowering effects. Collectively, these physiological effects of estrogen are significant in alleviating hypertension.

### 5.3. Clinical Research Findings on Estrogen Therapy for Cardiovascular Disease

For decades, it was a widely held belief that estrogen could protect perimenopausal women from the onset of cardiovascular disease. Two decades ago, however, the landmark Women’s Health Initiative (WHI) study cast doubt on this assumption. The study demonstrated that the combined use of estrogen and progestin was associated with a greater risk of adverse health outcomes than benefits, suggesting that this regimen should not be initiated or continued for the prevention of coronary heart disease [150]. A subsequent systematic review and meta-regression analysis of clinical trials of hormone therapy from 1979 to 2017 further refined this understanding. The review concluded that while hormone therapy initiated within 10 years of menopause may confer cardioprotective benefits, initiation beyond this window could potentially increase cardiovascular risk [151]. It appears that there exists a potential avenue for mitigating the risk of cardiovascular disease among menopausal women through the administration of estrogen supplements during this critical period. However, it is regrettable that the present review does not explicitly address the optimal dosage, frequency, method of administration, and duration of estrogen supplementation. This absence of detailed guidance leaves clinicians informed about the cardiovascular risk reduction associated with estrogen supplementation during this timeframe, yet without specific instructions regarding its utilization.

## 6. Osteoporosis

### 6.1. Estrogen and Osteoporosis

The relationship between estrogen and osteoporosis is well documented, particularly during perimenopause and menopause. The decline in estrogen levels during these stages contributes significantly to the increased prevalence of osteoporosis, as evidenced by studies [152,153,154]. Osteoporosis is primarily due to a decrease in bone formation coupled with an increase in bone resorption [7]. The primary cause of osteoporosis is a reduction in bone formation accompanied by an increase in bone resorption. This imbalance is characterized by a decrease in the number of osteoblasts, which are responsible for bone formation, and an increase in the number of osteoclasts, which are responsible for bone resorption. Estrogen plays a pivotal role in regulating both processes [7]. Consequently, estrogen replacement therapy (ERT) is prescribed for postmenopausal women with osteoporosis due to estrogen loss [155,156].

### 6.2. Estrogen’s Impact on Osteoporosis

**Figure 5:**
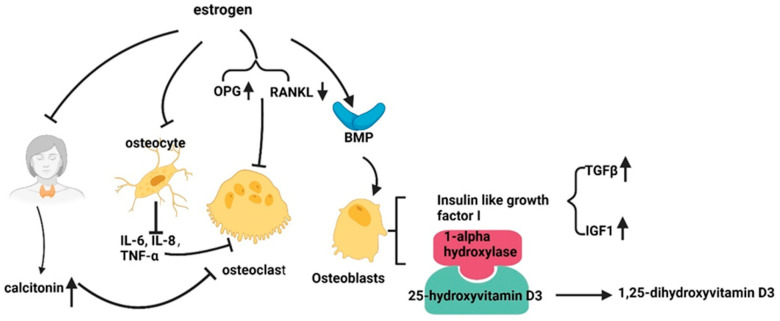
Schematic of how estrogen relieves osteoporosis. RANKL, receptor activator of NF Kappa; OPG, osteoprotegerin; IGF1, insulin-like growth factor I; TGFβ, transforming growth factor-β; ↑, promote; ↓, decrease; sharp arrows (→), stimulate; blunt arrows (┴), inhibit.

A substantial file of experimental evidence now supports the hypothesis that estrogen is a contributing factor in the development of osteoporosis. These effects are mediated through various mechanisms. As illustrated in Figure 5, estrogen exerts its effects on bone metabolism through a variety of mechanisms.

#### 6.2.1. Promote Osteogenesis

Estrogen facilitates the differentiation and formation of osteoblasts through several mechanisms. Firstly, it upregulates bone morphogenetic protein (BMP) signaling [7], which is essential for osteoblast differentiation and bone formation. Additionally, estrogen stimulates osteoblasts to produce insulin-like growth factor I (IGF1) and transforming growth factor-β (TGFβ), both of which promote bone formation and remodeling. Moreover, estrogen enhances the activity of 1-alpha hydroxylase, an enzyme responsible for converting 25-hydroxyvitamin D3 to its active form, 1,25-dihydroxy vitamin D3. This active form of vitamin D plays a crucial role in regulating calcium and phosphate metabolism, thereby supporting bone health [157].

#### 6.2.2. Inhibit Osteoclasts

The role of estrogen in osteoclasts is a topic of significant interest in the field of bone research. Estrogen plays a critical role in inhibiting the formation and differentiation of osteoclasts through several mechanisms. Firstly, it suppresses the expression of receptor activator of NF-kB ligand (RANKL), a key factor that activates osteoclasts, while promoting the production of osteoprotegerin (OPG), which acts as a decoy receptor for RANKL and, thus, inhibits osteoclast formation [158]. In addition, estrogen inhibits the secretion of bone resorption factors such as IL-1, IL-6, and TNF-α by osteoblasts, thereby indirectly inhibiting osteoclast differentiation [7,159]. Moreover, estrogen was found to exert a suppressive effect on the formation of osteoclast-like cells in response to parathyroid stimulation [160]. Furthermore, it stimulates the synthesis and secretion of calcitonin, a hormone that inhibits osteoclast function [161]. These combined actions contribute to the overall inhibition of osteoclast formation and activity by estrogen, thereby supporting bone health.

In conclusion, estrogen is involved in both the osteogenesis processes and osteoclasts, which collectively contribute to the prevention of osteoporosis.

### 6.3. Clinical Findings on the Efficacy of Estrogen in the Treatment of Osteoclast

Clinical research on the effectiveness of estrogen in the treatment of osteoporosis has yielded encouraging results. The Women’s Health Initiative (WHI) have indicated that estrogen therapy may preserve bone density and reduce the risk of fractures in postmenopausal women [162]. The initiation of estrogen therapy in the proximity of menopause appears to offer the most significant protective effects against osteoporosis-related fractures [163]. Further support for these findings is provided by observational studies [150] and meta-analyses [164,165], which demonstrate improvements in bone mineral density and a decreased fracture risk with estrogen therapy. In conclusion, these findings suggest that estrogen therapy may be an effective approach for maintaining skeletal health and preventing osteoporosis-related complications in menopausal women.

## 7. Gastrointestinal Diseases

### 7.1. Estrogen and Gastrointestinal Diseases

In recent years, researchers have established a correlation between sex hormones and the development of gastrointestinal diseases. A synthesis of findings from numerous studies indicates that females exhibit a lower prevalence of conditions such as irritable bowel syndrome (IBS) [166,167,168], gastroesophageal reflux disease (GERD) [169], and peptic ulcers [170] compared to males. This observation underscores the potential influence of estrogen in regulating the pathogenesis of digestive system diseases.

### 7.2. Estrogen’s Impact on Gastrointestinal Diseases

**Figure 6:**
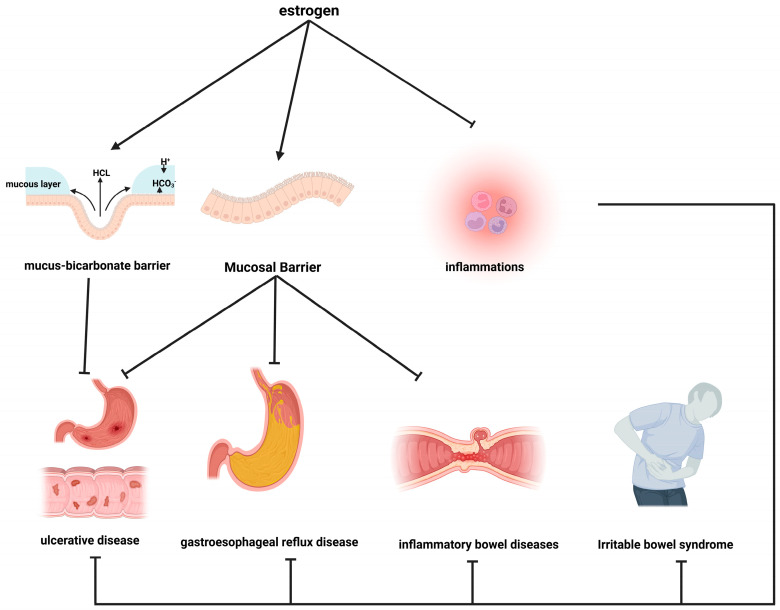
Schematic representation of estrogen action in gastrointestinal disorders; sharp arrows (→), stimulate; blunt arrows (┴), inhibit.

An increasing body of evidence suggests that estrogen plays a role in the modulation of gastrointestinal diseases affecting the esophagus, stomach, and intestines through a range of mechanisms, as illustrated in Figure 6.

#### 7.2.1. Maintain the Mucus–Bicarbonate Barrier

The mucus–bicarbonate barrier is a protective mechanism that forms a barrier between the gastric mucosa and gastric acid [171]. It is composed of a mucous layer that covers the gastric mucosa and a secreted layer of bicarbonate (HCO^3−^), which forms a barrier against gastric acid and pepsin damage [172]. It has been demonstrated that estrogen can regulate the secretion of HCO^3−^, which consequently reduces the likelihood of developing duodenal ulcers in females [173]. The precise mechanisms remain unclear, but research indicates that estrogen may stimulate HCO^3−^ secretion via intracellular calcium, cystic fibrosis transmembrane conductance regulator, and ER-dependent mechanisms related to Cl^−^/HCO^3−^ anion exchangers [174]. Furthermore, estrogen has also been shown to reduce basal acid secretion and quality, thereby conferring protective effects on the gastrointestinal tract [175].

#### 7.2.2. Strengthen the Epithelial Barrier

The epithelial barrier in the gastrointestinal tract plays a pivotal role in maintaining a sterile internal environment and preventing the infiltration of external contaminants. This is primarily achieved through tight junctions at the basal level [176]. Damage to the mucosal barrier is a significant contributing factor to the onset of conditions such as GERD [177], gastrointestinal ulcers [178], and inflammatory bowel disease (IBD) [179]. Estrogen plays a crucial role in maintaining the integrity of the gastrointestinal epithelial barrier. A study has shown that estrogen activates ERβ, leading to increased expression of proteins crucial for epithelial cell closure, such as junctional adhesion molecule-A (JAM-A) [180]. This activation enhances the barrier function of the intestinal epithelium. Furthermore, estrogen exerts a protective influence on the esophagus by mitigating transmembrane resistance and reducing epithelial permeability induced by esophageal stimuli. This is achieved by upregulating the expression of occludin, a tight junction protein that enhances junctional adhesion and reinforces esophageal barrier function [181]. Therefore, estrogen’s role in fortifying the epithelial barrier not only benefits the esophagus but also contributes to gastrointestinal health.

#### 7.2.3. Modulate Inflammation

Recurrent inflammation has been demonstrated to markedly enhance the susceptibility to conditions such as GERD [182], peptic ulcers [183], IBS [184], and IBD [185]. Estrogen plays a pivotal role in the regulation of inflammation within the gastrointestinal tract [8]. Its anti-inflammatory properties contribute to the mitigation of tissue damage in GERD [186] and the alleviation of abdominal discomfort associated with both IBS and IBD [187]. Studies indicate that estrogen has the capacity to reduce mast-cell-mediated toxicity and inhibit the release of the inflammatory cytokine TNF-α, which serves to protect esophageal tissues from damage [188]. Helicobacter pylori is among the most prevalent chronic bacterial infections in humans, affecting approximately 90% of individuals diagnosed with duodenal ulcers and approximately 80% of those with gastric ulcers [189]. The capacity of estrogen to impede the adhesion of Helicobacter pylori suggests a potential prophylactic strategy against the formation of peptic ulcers [190]. In IBD, the anti-inflammatory mechanism of estrogen may involve ERβ activation, which reduces intracellular calcium levels in macrophages. This prevents the assembly of NLR family pyrin domain containing 3 (NLRP3) inflammasomes and limits the production of IL-1β [191].

Therefore, estrogen’s function in reinforcing the gastrointestinal epithelial barrier, maintaining the equilibrium of physiological barriers, and reducing inflammatory responses highlights its protective role in the digestive system. This multifaceted action offers potential for mitigating the onset of GERD, gastrointestinal ulcers, IBS, and IBD.

### 7.3. Clinical Findings on the Efficacy of Estrogen in the Treatment of Gastrointestinal Diseases

Despite the aforementioned exploration of potential protective mechanisms of estrogen in the gastrointestinal system, clinical evaluations regarding the efficacy of estrogen therapy for gastrointestinal diseases have yielded inconsistent results, varying among specific conditions. For example, in the context of GERD, women undergoing estrogen therapy exhibited a trend towards a higher incidence of symptomatic GERD (4.2%) compared to those receiving a placebo (3.1%) [192]. Clinical trials assessing the efficacy of estrogen therapy for gastrointestinal ulcers have been notably limited. A report dating back to 1968 suggested that estrogen therapy might reduce ulcer size; however, due to the small number of patients, this difference was not statistically significant, and the results cannot be considered conclusive [193]. A different finding has been observed in the evaluation of estrogen therapy for IBS, whereby hormone treatment has been found to increase the risk of developing IBS [194]. Moreover, findings from a large prospective cohort study indicated an association between postmenopausal hormone replacement therapy and an increased risk of ulcerative colitis (UC), but not with Crohn’s disease (CD) [195]. These findings underscore the complexity and variable effects of estrogen therapy in different gastrointestinal conditions, and further research is needed to provide clearer clinical guidance.

## 8. Estrogen-Dependent Cancers

### 8.1. Estrogen and Estrogen-Dependent Cancers

Despite its pivotal role in regulating a multitude of systemic diseases, estrogen is also regarded as a carcinogen. This suggests that estrogen may contribute to the development of estrogen-dependent cancers, including breast, ovarian, and endometrial cancers. The progression and growth of these cancers is typically dependent on the presence of estrogen. Consequently, the most common treatment strategies entail the inhibition of estrogen production within the body or the prevention of estrogen from binding to its receptors. The current literature indicates that the expression of ERα may facilitate the progression of breast [196], ovarian [197], and endometrial cancers [198], whereas ERβ exhibits the opposite effect [199,200,201]. Despite inconsistent effects of GPER on these cancers, studies have shown that overexpression of GPER is associated with poor prognosis in these cancers [202]. A deeper understanding of the mechanisms by which estrogen-dependent cancers act through specific signaling pathways of different estrogen receptors is helping to develop drugs that target these pathways, potentially leading to more effective treatments for estrogen-dependent cancers and greater benefits for women’s health.

### 8.2. Estrogen’s Impact on Estrogen-Dependent Cancers

**Figure 7:**
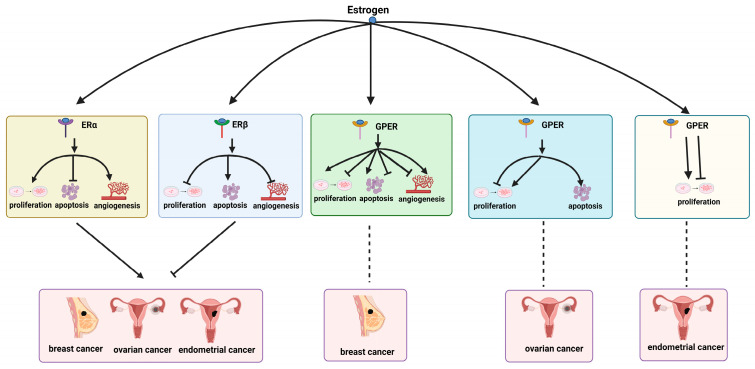
Schematic representation of estrogen action in estrogen-dependent cancers. ER, estrogen receptor, GPER, G protein-coupled estrogen receptor; sharp arrows (→), stimulate; blunt arrows (┴), inhibit; ﹍, stimulate or inhibit.

Estrogen exerts its regulatory influence over the biological processes associated with estrogen-dependent cancers through its binding to a range of estrogen receptors. Subsequently, these complexes modulate cancer cell proliferation, apoptosis, and angiogenesis, thereby influencing cancer development and growth (see Figure 7).

#### 8.2.1. Regulate Cell Proliferation

The term “cell proliferation” is used to describe the rate at which cancer cells replicate their DNA and divide into two daughter cells. This process is considered a critical component of cancer development and progression [203]. A substantial body of research demonstrates that estrogen exerts intricate regulatory influences on cancer cell proliferation through its binding to diverse types of estrogen receptors [204,205].

In breast cancer cells, ERα has been demonstrated to promote cell proliferation through different pathways. For example, it may enhance protein expression, such as that of ubiquitination [206], activate glycogen synthase kinase-3 beta (GSK3β) [207], increase levels of proliferating cell nuclear antigen (PCNA) and marker of proliferation (Ki-67) [208], and also regulate the cell cycle via the phosphatidylinositol 3-kinase (PI3K)/protein kinase B (AKT) pathway [209,210], thereby promoting breast cancer cell proliferation. In ovarian cancer cells, ERα may induce the expression of semaphorin (Sema) 4D [211], influencing the cell cycle progression involving cellular-fos (c-fos), cellular Myc (c-myc), growth factors, and cell cycle proteins to promote cell proliferation [212]. Conversely, the inhibition of ERα expression may result in the upregulation of peroxisome proliferator-activated receptor gamma (PPARγ), which, in turn, may lead to the inhibition of proliferation in endometrial cancer cells [213].

In comparison to ERα, ERβ has been demonstrated to exert an inhibitory effect on the proliferation of estrogen-dependent tumors. ERβ achieves this by promoting mitofusin 2 (MFN2) gene expression [214], and inducing G2 cell cycle arrest, thereby inhibiting the proliferation of breast cancer cells [199]. Moreover, ERβ has been observed to suppress the expression of genes such as sema 4D [211], retinoblastoma protein (Rb), phosphorylated (p)-Rb, and AKT, which, in turn, reduces the frequency of S-phase cells and increases G2/M-phase cells [215], ultimately leading to the inhibition of proliferation in ovarian cancer cells. Conversely, the literature indicates that the downregulation of ERβ signaling may also promote proliferation in endometrial cancer, although the specific mechanisms remain unclear [216].

In estrogen-dependent cancers, the role of GPER in cancer cell proliferation appears to be inconsistent across different types of cancer. In breast cancer, GPER promotes cell proliferation by targeting miR-124/cluster of differentiation 151 (CD151) [217] and enhancing the expression of cancer-associated fibroblast (CAF) proteins [218]. Conversely, another study indicates that GPER activation can inhibit breast cancer cell proliferation by reducing cyclin B expression and inducing cell cycle arrest at the G2/M phase [219]. Similarly, conflicting results are observed regarding the proliferative effects of GPER on ovarian cancer cells. Some studies suggest that activating GPER increases the number of cells in the S phase of the cell cycle and upregulates levels of cyclin D1 and c-fos to promote cell proliferation [220]. In contrast, another study suggests that GPER activation inhibits ovarian cancer cell proliferation by blocking microtubule protein aggregation [221]. The role of GPER in promoting proliferation in endometrial cancer is also uncertain. A study indicates that GPER enhances the proliferation of endometrial cancer cells, primarily through mechanisms involving the regulation of c-fos and cyclin D1 production [222]. However, there is also report suggesting that activation of GPER can lead to inhibition of endometrial cancer cells [223].

#### 8.2.2. Regulate Apoptosis

In the context of estrogen-dependent cancer cells, the regulation of apoptosis by estrogen represents a pivotal target for cancer therapy [224]. The effects of estrogen on apoptosis in cancer cells are complex and diverse, occurring through binding to different types of estrogen receptors.

In particular, ERα frequently displays anti-apoptotic properties in estrogen-dependent cancers. For example, in breast cancer cells, ERα can inhibit p53-mediated transcriptional activation and prevent p53-dependent cell apoptosis [225]. In contrast, in ovarian cancer, estrogen alpha receptor inhibitor MPP has been observed to promote the expression of apoptotic proteins, including caspase-3 and Bax, and to enhance the activity of the cancer suppressor gene p53 [226]. This has been demonstrated to influence the balance of apoptotic regulation in cells. Nevertheless, in endometrial cancer cells, the activation of ERα signaling pathways may inhibit apoptosis by causing p-Akt translocation into the nucleus [227].

In contrast to the role of ERα, ERβ has been shown to play a pro-apoptotic role in the development of these types of cancers. Research indicates that grape seed extract can promote apoptosis in breast cancer cells by increasing the expression of estrogen receptor beta (ERβ) as well as apoptosis-related genes such as Annexin V protein and caspase-3 [228]. In ovarian cancer cells, the activation of ERβ has been demonstrated to significantly reduce cell viability and promote apoptosis [229,230]. Furthermore, transfection of endometrial cancer cells with estrogen receptor 2 (ESR2) siRNA has been demonstrated to result in a significant downregulation of the apoptosis-related gene TATA-box binding protein-associated factor 9B (TAF9B), thereby confirming the positive role of ERβ in apoptosis regulation [201].

With respect to GPER, studies of ovarian and endometrial cancer have demonstrated its involvement in the promotion of cell apoptosis. Nevertheless, its function in apoptosis regulation in breast cancer remains a topic of contention. Some studies have proposed that GPER may regulate apoptosis in breast cancer cells by upregulating apoptotic signaling pathways [231], whereas other studies have indicated that it may inhibit apoptosis by reducing the expression of caspase 3 and caspase 7 through the PI3K/AkT pathway [232]. In ovarian cancer, GPER functions as a fatty acid receptor mechanism involving increased expression of apoptotic proteins such as B-cell lymphoma 2-like protein 11 (Bim) and Bax, and promotion of protein kinase A (PKA) activity and cyclic adenosine monophosphate (cAMP) levels. This ultimately induces apoptosis in ovarian cancer cells [233]. A study has demonstrated that its agonist G1 can induce apoptosis in endometriotic stromal cells via a caspase-3-dependent pathway, exhibiting minimal cytotoxicity [234]. However, the precise mechanism through which GPER regulates apoptosis in endometrial cancer cells remains unclear.

#### 8.2.3. Regulate Angiogenesis

Cancer angiogenesis, defined as the process of generating new blood vessels from existing vessels surrounding caners, plays a pivotal role in the development of cancer [235]. 

ERα has been demonstrated to promote angiogenesis in these cancers. For example, shERα cells have been observed to markedly diminish the expression of angiogenic molecular markers, including vascular endothelial growth factor A (VEGF-A) and angiopoietin-2 (Ang-2), in breast cancer cells. This has been demonstrated to impede the formation and growth of new blood vessels [236]. In ovarian cancer cells, estrogen has also been demonstrated to promote the expression of angiogenic factors such as nerve growth factor (NGF) and vascular endothelial growth factor (VEGF) through the action of ERα [237]. By contrast, the inverse agonist XCT790 affects the process in endometrial cancer cells by inhibiting the transcriptional activity of VEGF induced by ERα [238].

In contrast, ERβ functions in opposition to ERα in these cancers. Agonists of ER β have been demonstrated to induce the expression of ERβ, thereby reducing the expression of angiogenic markers in breast cancer [239]. In ovarian cancer cells, the metastasis-associated gene methionine adenosyltransferase 1 (MTA1) has been observed to reduce the expression of ERβ while simultaneously increasing the expression of the angiogenic cytokine growth-regulated oncogene (GRO) [240]. In endometrial cancer cells, the ERβ antagonist PHTPP has been demonstrated to reverse estrogen-induced angiogenesis [241].

The role of GPER in breast cancer angiogenesis remains inconclusive in the existing literature. Some studies suggest that 17β-estradiol (E2) and the GPER-selective agonist G-1 may upregulate hypoxia-inducible factor 1-alpha (HIF1α) through the GPER/epidermal growth factor receptor (EGFR)/extracellular signal-regulated kinase (ERK)/c-fos signaling pathway, thereby increasing VEGF expression [241,242]. However, alternative research suggests that in triple-negative breast cancer (TNBC), activation of GPER inhibits TNBC migration and angiogenesis by blocking the NF-κB/P65 signaling pathway and reducing expression of the endothelial marker CD34 [243]. At present, there is limited research in the literature that explicitly reports whether GPER is involved in the angiogenesis processes of ovarian cancer or endometrial cancer. 

Based on the above analysis, estrogen primarily regulates estrogen-dependent cancers such as breast, ovarian, and endometrial cancers by modulating cancer cell proliferation, apoptosis, and angiogenesis.

### 8.3. Clinical Findings of Estrogen Therapy for Estrogen-Dependent Cancer

As research continues to delve deeper into the effects of estrogen, more and more clinical trials are revealing its effects on estrogen-dependent cancers. In breast cancer, the majority of studies indicate that estrogen therapy does not significantly increase the risk of developing breast cancer [244,245,246,247], although some suggest a slight increase in risk [248]. Similarly, studies of endometrial cancer show that estrogen therapy does not increase the risk in women [249,250]. Nevertheless, studies on ovarian cancer indicate that women who have previously undergone estrogen therapy exhibit a markedly elevated risk compared to those who have not [251]. These findings underscore the complex role of estrogen in the development of different types of cancers. While estrogen therapy appears to be relatively safe for breast and endometrial cancers, concerns about an increased risk of ovarian cancer are troubling. Therefore, when considering estrogen therapy, it is important to thoroughly evaluate individual factors and potential tumor risks through a detailed risk assessment.

## 9. Discussion and Conclusions

**Figure 8:**
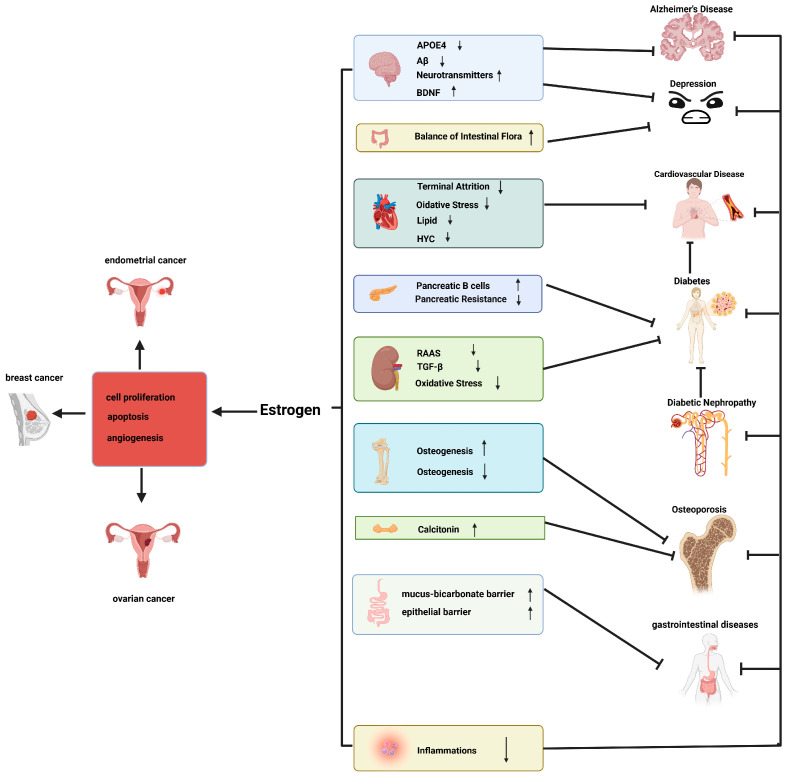
Schematic of the actions of estrogen in multiple diseases; ↑, promote; ↓, decrease; sharp arrows (→), stimulate; blunt arrows (┴), inhibit.

Estrogen is an important sex hormone that plays a pivotal role in regulating numerous physiological processes within the human body. This review examines the mechanisms of estrogen action in a multitude of diseases, including AD, depression, cardiovascular disease, diabetes mellitus, osteoporosis, gastrointestinal diseases, and estrogen-dependent cancers. Furthermore, it presents a summary of the efficacy of estrogen therapy for these diseases in clinical practice. Our findings indicate that estrogen plays a role in regulating the onset and progression of these diseases through various pathways, including effects on neuronal protection, mood regulation, vascular function, insulin sensitivity, bone metabolism, gastrointestinal barrier function, and the proliferative role of cancer cells. Of particular note is the observation that when the mechanisms by which estrogen acts on these diseases are connected (Figure 8), it becomes evident that estrogen regulates inflammatory factors and simultaneously interferes with the onset and progression of most of diseases. Although this phenomenon is of great interest, there is currently no research in the literature on whether there is organ variability in the regulation of inflammatory factors by estrogen and differences in the channels they regulate. Future studies could examine this direction in order to delve into the mechanisms of estrogen regulation of inflammation in various organs, thus realizing the possibility of a multidrug effect.

Furthermore, this review offers a concise summary of the impact of estrogen therapy on a range of medical conditions. However, the constraints of space preclude a detailed elaboration and analysis of these effects. While basic research has demonstrated the efficacy of estrogen for these diseases, preliminary findings suggest that there is variation and uncertainty in the efficacy of estrogen therapy for various diseases. Furthermore, there is an increased risk of estrogen-dependent cancers. Moreover, a review of the literature on this type of clinical research revealed a paucity of studies, resulting in an absence of definitive guidelines for the utilization of estrogen in these diseases. While basic research has intrinsic value, clinical studies represent the gold standard for validating the effectiveness of such findings. In light of the aforementioned understanding of the mechanisms of estrogen action in a wide range of diseases, it is recommended that further clinical trials of estrogen therapy be conducted for the benefit of older women.

## Data Availability

Not applicable.
